# Are radiomic spleen features useful for assessing the differentiation status of advanced gastric cancer?

**DOI:** 10.3389/fonc.2023.1167602

**Published:** 2023-05-05

**Authors:** Dongbo Lyu, Pan Liang, Chencui Huang, Xingzhi Chen, Ming Cheng, Bingbing Zhu, Mengru Liu, Songwei Yue, Jianbo Gao

**Affiliations:** ^1^ The Departments of Radiology, the First Affiliated Hospital of Zhengzhou University, Zhengzhou, China; ^2^ The Department of Research Collaboration, R&D Center, Beijing Deepwise & League of PHD Technology Co., Ltd, Beijing, China; ^3^ The Departments of Information Department, the First Affiliated Hospital of Zhengzhou University, Zhengzhou, China

**Keywords:** spleen, differentiation, radiomics, tomography, x-ray computed, gastric cancer

## Abstract

**Background:**

The differentiation status of gastric cancer is related to clinical stage, treatment and prognosis. It is expected to establish a radiomic model based on the combination of gastric cancer and spleen to predict the differentiation degree of gastric cancer. Thus, we aim to determine whether radiomic spleen features can be used to distinguish advanced gastric cancer with varying states of differentiation.

**Materials and methods:**

January 2019 to January 2021, we retrospectively analyzed 147 patients with advanced gastric cancer confirmed by pathology. The clinical data were reviewed and analyzed. Three radiomics predictive models were built from radiomics features based on gastric cancer (GC), spleen (SP) and combination of two organ position (GC+SP) images. Then, three Radscores (GC, SP and GC+SP) were obtained. A nomogram was developed to predict differentiation statue by incorporating GC+SP Radscore and clinical risk factors. The area under the curve (AUC) of operating characteristics (ROC) and calibration curves were assessed to evaluate the differential performance of radiomic models based on gastric cancer and spleen for advanced gastric cancer with different states of differentiation (poorly differentiated group and non- poorly differentiated group).

**Results:**

There were 147 patients evaluated (mean age, 60 years ± 11SD, 111 men). Univariate and multivariate logistic analysis identified three clinical features (age, cTNM stage and CT attenuation of spleen arterial phase) were independent risk factors for the degree of differentiation of GC (*p* =0.004,0.000,0.020, respectively). The clinical radiomics (namely, GC+SP+Clin) model showed powerful prognostic ability in the training and test cohorts with AUCs of 0.97 and 0.91, respectively. The established model has the best clinical benefit in diagnosing GC differentiation.

**Conclusion:**

By combining radiomic features (GC and spleen) with clinical risk factors, we develop a radiomic nomogram to predict differentiation status in patients with AGC, which can be used to guide treatment decisions.

## Introduction

Gastric cancer (GC) occupies the fifth place among malignant tumors and the fourth place among cancer mortality rates, according to the latest global cancer burden data (Globocan 2020) (1). Furthermore, according to data from the report of Feng et al. (2), there are significant associations between differentiation status and tumor depth, lymph node metastasis, and tumor stage in GC. Other studies have confirmed that the five-year survival rates of advanced GC with different differentiation states are different, which is an independent risk factor affecting prognosis (3, 4). Thus, GC differentiation plays a crucial role in determining the surgical approach and prognosis of patients.

As a noninvasive examination method, computed tomography (CT) is commonly used to diagnose, evaluate, and treat patients with GC (5, 6). However, during the CT examination, small tumors or poor gastric dilatation may affect the diagnostic accuracy, On the other hand, unlike parenchymal organs, stomach is a hollow organ, and it is difficult to obtain accurate data for tumor delineation (7). With the development in recent years, dual-energy CT can be used as a supplement to conventional CT to improve the diagnostic efficiency, but its sensitivity (53%-85%) and specificity (85%-93%) in determining the differentiation status of GC are still insufficient, and Dual-energy CT requires a high degree of expertise of imaging technicians, which makes it difficult to popularize this scanning technology in primary hospitals (8–10). It is therefore necessary to develop an effective predictor model based on imaging data of non-tumor tissues. Among peripheral lymphoid organs, the spleen is the largest and most important immune organ outside the Tumor microenvironment (11). And spleens contain large numbers of B and T lymphocytes, which play a vital role in defending against tumors (12). Based on previous researches, GC patients who take a herb formula for rejuvenating the spleen have a better prognosis and a longer overall survival rate (13–15). Besides, some scholars found that diffuse reduction of spleen density is an independent predictor of post-operative outcomes after curative gastrectomy in patients with GC (16). Zhang’s study shows that patients with low splenic density have more advanced tumors and a poor prognosis, but they benefit more from chemotherapy (17).

Radiomics is a new method of diagnosing and predicting health conditions, which can easily identify heterogeneity within tissues and uses an automatic high-throughput feature data extraction algorithm to convert image data into data with high-resolution readability (18–20). The use of radiomics for the diagnosis and classification of GC, as well as for predicting prognosis and evaluating treatment efficacy, has been widely adopted (21–25). It has been proved that the differentiation degree of gastric cancer can be predicted based on the CT texture features of gastric cancer, and the AUC is 0.77 (26). Up to now, literature reports have been published relating to predicting the prognosis of GC patients with spleen radiomics, but these studies were only based on the spleen and not combined with the tumor lesions (7, 16). The purpose of this study was to explore the use of imaging data to explore the correlation between spleen-related characteristics and the prognosis of GC by establishing a more accurate and adaptable prognostic model. This study aimed to establish a radiomic model of GC and spleen so that the degree of differentiation of GC can be predicted more accurately, and to help clinical treatment decision.

## Materials and methods

### Patients

**Figure 1:**
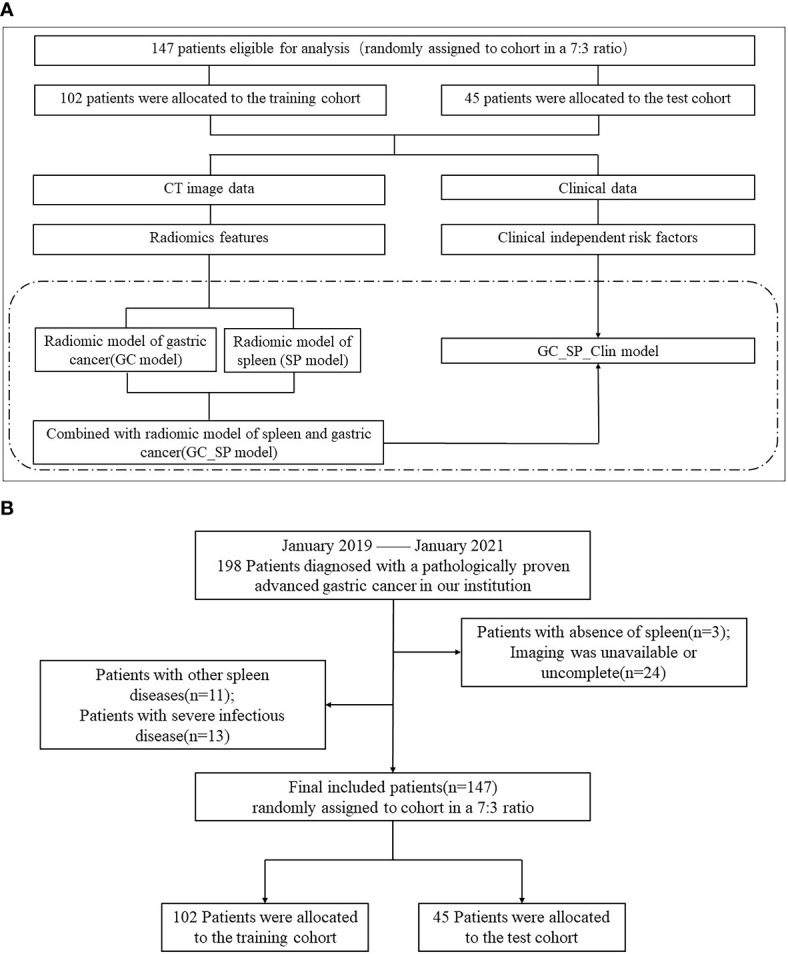
Flowchart of study and patient selection. **(A)** The study flowchart (The study mainly compared the four models in the dotted box). **(B)** The patient selection flowchart.

This study was approved by the institutional review board (No. 2021-KY-1070-002), and the requirement for written informed consent was waived in this retrospective study. 198 Patients with advanced GC confirmed by pathology in the first affiliated hospital of Zhengzhou university from January 2019 and January 2021 were collected retrospectively. All patients were subjected to a preoperative CT scan in 30 days. The inclusion criteria were as follows: (A) diagnosis of advanced GC based on postoperative pathology, (B) patients undergoing surgical dissection, and (C) complete clinical data. We excluded the following patients: (A) absence of spleen, (B) whose imaging data was unavailable or uncomplete, (C) with other spleen diseases, and (D) with severe infectious disease. Ultimately, 147 patients were included in this study and divided them into two groups in a 7:3 ratio: the training cohort (102 patients) and the test cohort (45 patients). The study flowchart and patient selection flowchart were shown in **Figure 1**.

### Data collection

The following data were collected in our hospital (1): clinicopathological features, including age, sex, and other chronic Diseases (eg: diabetes, hypertension, etc) (2); tumor features, including size, location, CT attenuation, Borrmann type, cT (clinical Tumor) stage, cN (clinical Node) status and cTNM (clinical Tumor node metastasis) stage (3); Biochemical indicators, including TAP(Tumor abnormal protein), CEA(a Carcinoma Embryonic Antigen), AFP(Alpha-fetoprotein) and CA19-9 (4); immune-related serological indicators, including WBC(White Blood Cell Count), PLT (Platelet Count), PLR (Platelet Lymphocyte Ratio) and NLR(Neutrophil Lymphocyte Ratio); and (5) spleen features, including thickness, and CT attenuation. Details are explained in **Appendix E1**.

In addition, two radiologists (with five and ten years experiences in gastroenterology imaging, respectively), who were blinded to the clinicopathological information, interpreted CT images independently and determined the state of differentiation according to the way the lesions were enhanced on CT images (26). In the event of a discrepancy in results, the decision is made by consultation between the two radiologists.

### Image acquisition and segmentation

All patients underwent an abdominal enhancement CT scan within 30 days of preoperation, covering the entire spleen. The CT examinations were conducted using a 64 multidetector CT scanner (Discovery CT750HD, GE Healthcare, Wisconsin, USA). A conventional axial scan (120 kV, 350 mA, a field of view = 500 mm, matrix 512×512, and section thickness 5 mm) was performed. Details are explained in **Appendix E1**.

CT image segmentation was carried out with section thickness 5 mm. A radiologist with 5 years of experience in gastrointestinal radiology interpreted CT enhanced venous phase images and used ITK-SNAP version 3.6.0 software to delineate regions of interest (ROIs) and implement manual tumor segmentation. Details are explained in **Appendix E1** and **Figure E1, E2.**

After one month, 15 patients were selected randomly and re-segmented by the radiologist to achieve high intra-rater agreement for radiomics analysis.

### Extraction of radiomics features

Resanmpling was performed to avoid data heterogeneity bias. And Laplacian of Gaussian (LoG), Local Binary Pattern (LBP) 2D, LBP3D, wavelet, square, squareroot, logarithm, exponential and gradient filtering were conducted for imaging transformation. Radiomics features were calculated from ROIs of original images and transformed images respectively *via* the Deepwise Multimodal Research Platform (https://keyan.deepwise.com, v2.0), including first-order features, shape features and texture features including gray-level size zone matrix (GLSZM), gray-level run length matrix (GLRLM), gray level co-occurrence matrix (GLCM), gray-level diference matrix (GLDM) and neighboring gray tone difference matrix (NGTDM).

### Feature reduction and radiomics signature construction

All features were scaled using Z-standardisation (X−X/SD). Intraclass correlation coefficient analysis (ICC) was conducted to slash unstable radiomics features owing to artificial segmentation. A total of 15 cases were randomly selected for SP groups and GC groups respectively to delineate ROIs again by the same radiologist. Features with high stability(ICC≥0.75) were selected for further feature selecting. Then, one of the features will be removed to alleviate the redundancy *via* feature correlation analysis. Finally, F test was conducted to screen features further. The remaining features was used for radiomics signature construction of GC model and SP model. In order to verify the stability of the established final combined radiomics nomogram model, in addition to the conventional test cohort, we carried on five-fold cross validation strategy based on all the data of 147. All data was divided into a training set and a test set by splitting the dataset randomly into five groups, and one group was used as a test cohort while the remaining four groups were used as a training cohort. This procedure was repeated 5 times.

### CT–Based nomogram construction and evaluation

The weighted sum of radiomics features with non-zero coeffificients in SP group and GC group yielded the SP model and GC model respectively. And GC+SP model was yielded based on the selected features from features extracted from SP and GC groups.

Univariable and multivariable logistic regression was performed to select predictive clinical features. GC+SP+Clin model were developed incorporating the GC+SP model score and selected clinical features, which termed CT–Based nomogram model.

For comparison of performance of CT–Based nomogram model with the GC model, SP model and GC+SP model, AUC (95%CI), sensitivity, specificity, accuracy, F1-score and precision were calculated. ROC curve was also drawn to evaluate the classification ability of models. Decision curve was applied to analyze the clinical efficacy of different machine learning models. We also performed Delong test to calculate statistical differences of models.

### Statistical analyses

Statistical analysis was conducted using SPSS software (version 21.0, IBM), with *p*< 0.05 indicating a statistically significant difference. In univariate analyses, the continuous variables were analyzed for normality of the distribution by use of the Kolmogorov-Smirnov test. The Mann-Whitney U test or independent *t* test was used to compare differences in the continuous variables. The disordered classification variables were compared by Chi-square test or Fisher exact test. The ordered classification variables were tested by Kruskal Wallis test. Multiple analyses were performed using forward stepwise regression. The intra-class correlation coefficient was used to evaluate the consistency of radiomic parameters extracted by the same tester. ICC ≥ 0.75 was defined as consistent superiority. Features selection and modeling were conducted on the Deepwise Multimodal Research Platform v2.0(Beijing Deepwise & League of PHD Technology Co., Ltd, Beijing, China, https://keyan.deepwise.com), and Delong test was conducted *via* medcalc (version 11.4.2.0, https://www.medcalc.org).

## Results

### Clinical characteristics and CT parameters

In this study, 147 patients were eventually included, including 78 patients with poorly differentiation and 69 patients with non- poorly differentiation), 102 patients in the training cohort (54 patients with poorly differentiation and 48 patients with non- poorly differentiation), and 45 patients in the test cohort (24 patients with poorly differentiation and 21 patients with non- poorly differentiation). The clinical characteristics and CT parameters of patients in the training cohort and test cohort are summarized in **Table 1**. In the training cohort, there were significant differences in age, cT stage, cN, cTNM stage, CT attenuation of tumor arterial phase, CT attenuation of spleen arterial phase and Splenomegaly between the poorly differentiated group and the non-poorly differentiated group (all *p* < 0.05). And there was no statistically significant difference between the two groups in the remaining parameters. As for the test cohort, except for age, sex and tumor thickness, there were no statistically significant differences in other clinical and CT parameters. As shown in **Table 1**, parameters (*p* < 0.20) were screened in univariate analysis and multivariate logsitic analysis was performed. The results showed that age, cTNM stage and CT attenuation of spleen arterial phase were independent risk factors for the degree of differentiation of GC after adjustment for cofactors (*p* =0.004,0.000,0.020, respectively, as shown in **Table E1**).

**Table 1:** Clinical Characteristics and CT Parameters of Patients in Training and Test Cohorts.

Characteristic		Training cohort			p	Test cohort			p
		All (n =102)	Poorly (n=54)	Non poorly		All (n =45)	Poorly (n=24)	Non poorly	
				(n=48)				(n=21)	
Age		59.56 ± 11.98	56.87 ± 13.32	62.58 ± 9.53	0.010*	60.73 ± 9.25	57.75 ± 9.61	64.14 ± 7.68	0.020*
Gender									
Male		78 (76)	40 (74)	38 (79)	0.55	33 (73)	14 (58)	19 (90)	0.020*
Female		24 (24)	14 (26)	10 (21)		12 (27)	10 (42)	2 (10)	
Hepatitis									
No		100 (98)	52 (96)	48 (100)	0.18	44 (98)	24 (100)	20 (95)	0.28
Yes		2 (2)	2 (4)	0 (0)		1 (2)	0 (0)	1 (5)	
Borrmann type									
I		5 (5)	1 (2)	4 (8)		1 (2)	0 (0)	1 (5)	
II		22 (21)	12 (22)	10 (21)	0.27	9 (20)	3 (12)	6 (29)	0.1
III		59 (58)	30 (56)	29 (60)		24 (53)	12 (50)	12 (57)	
IV		16 (16)	11 (20)	5 (11)		11 (25)	9 (38)	2 (9)	
cT stage									
2		15 (15)	3 (6)	12 (25)		5 (11)	2 (8)	3 (14)	
3		41 (40)	18 (33)	23 (48)	0.001**	23 (51)	11 (46)	12 (57)	0.47
4		46 (45)	33 (61)	13 (27)		17 (38)	11 (46)	6 (29)	
Status cN									
N-		27 (26)	9 (17)	18 (38)	0.020*	15 (33)	5 (21)	10 (48)	0.057
N+		75 (74)	45 (83)	30 (62)		30 (67)	19 (79)	11 (52)	
cTNM stage									
I		6 (6)	2 (4)	4 (8)		4 (9)	2 (8)	2 (9)	
II		34 (33)	8 (15)	26 (54)	0.000**	13 (29)	3 (13)	10 (48)	0.040*
III		55 (54)	37 (68)	18 (38)		25 (55)	16 (66)	9 (43)	
IV		7 (7)	7 (13)	0 (0)		3 (7)	3 (13)	0 (0)	
**Tumor thickness (mm)**		23.49 ± 8.14	24.67 ± 8.72	22.16 ± 7.29	0.12	26.49 ± 9.45	29.12 ± 10.18	23.47 ± 7.71	0.040*
CT attenuation of GC (HU)									
unenhanced phase		36.73 ± 8.42	37.37 ± 8.54	36.00 ± 8.31	0.42	37.90 ± 7.28	37.57 ± 5.89	38.29 ± 8.73	0.74
arterial phase		69.81 ± 17.17	66.20 ± 16.74	73.88 ± 16.89	0.020*	74.77 ± 16.93	76.65 ± 18.45	72.61 ± 15.17	0.43
venous phase		80.94 ± 17.53	81.26 ± 18.57	80.58 ± 16.46	0.85	85.40 ± 16.29	86.80 ± 18.33	83.81 ± 13.87	0.54
Splenomegaly									
No		94 (92)	47 (87)	47 (98)	0.040*	43 (96)	22 (92)	21 (100)	0.18
Yes		8 (8)	7 (13)	1 (2)		2 (4)	2 (8)	0 (0)	
CT attenuation of spleen (HU)									
unenhanced phase		50.41 ± 5.27	50.29 ± 4.64	50.54 ± 5.94	0.81	51.89 ± 5.30	50.99 ± 5.49	52.93 ± 5.00	0.23
arterial phase		119.15 ± 22.82	114.01 ± 24.23	124.94 ± 19.80	0.020*	118.10 ± 28.07	121.26 ± 31.48	114.49 ± 23.85	0.43
venous phase		111.66 ± 16.42	111.94 ± 16.83	111.35 ± 16.11	0.86	114.01 ± 14.54	112.78 ± 13.23	115.42 ± 16.11	0.55

Unless indicated otherwise, the data presented are the number of patients, with percentages in parentheses. *p <0.05, **p <0.01, data are mean value ± SD; SD, standard deviation; cTNM stage, clinical tumor node metastasis stage; CT, computed tomography; GC, gastric cancer.

### Feature reduction and radiomics signature construction

**Figure 2:**
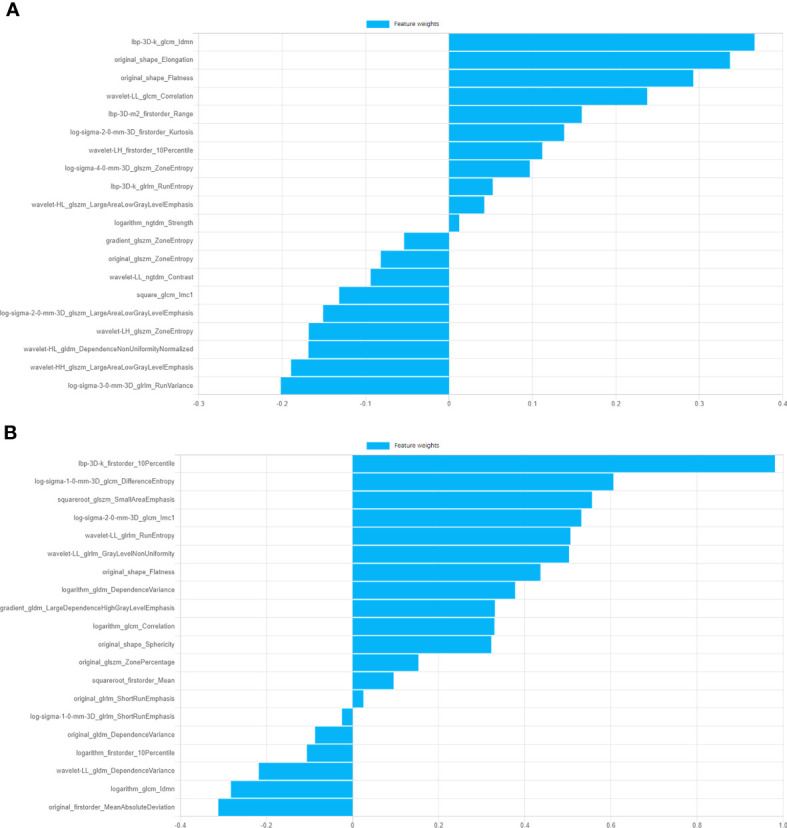
Information of top ten features of GC model and SP model and corresponding feature weights. **(A)** Features weights of GC model. **(B)** Features weights of SP model.

1743 features were extracted from ROIs of SP and GC groups respectively, 543 and 90 features were removed after ICC ananlysis (ICC ≥ 0.75) respctively. After feature correlation analysis and F test algorithm, 25 features were remained for SP group to establish SP model, including 5 first-order features, 2 shape features and 18 texture features(2 GLSZM, 5 GLRLM, 4 GLCM, 6 GLDM and 1 NGTDM)and 25 features were remained for GC group to establish GC model, including 4 first-order features, 2 shape features and texture features including 10 GLSZM, 2 GLRLM, 3 GLCM, 1 GLDM and 3 NGTDM. Ten of the most weighted features and their weights were showed in **Figure 2**.

### Nomogram construction and evaluation

**Figure 3:**
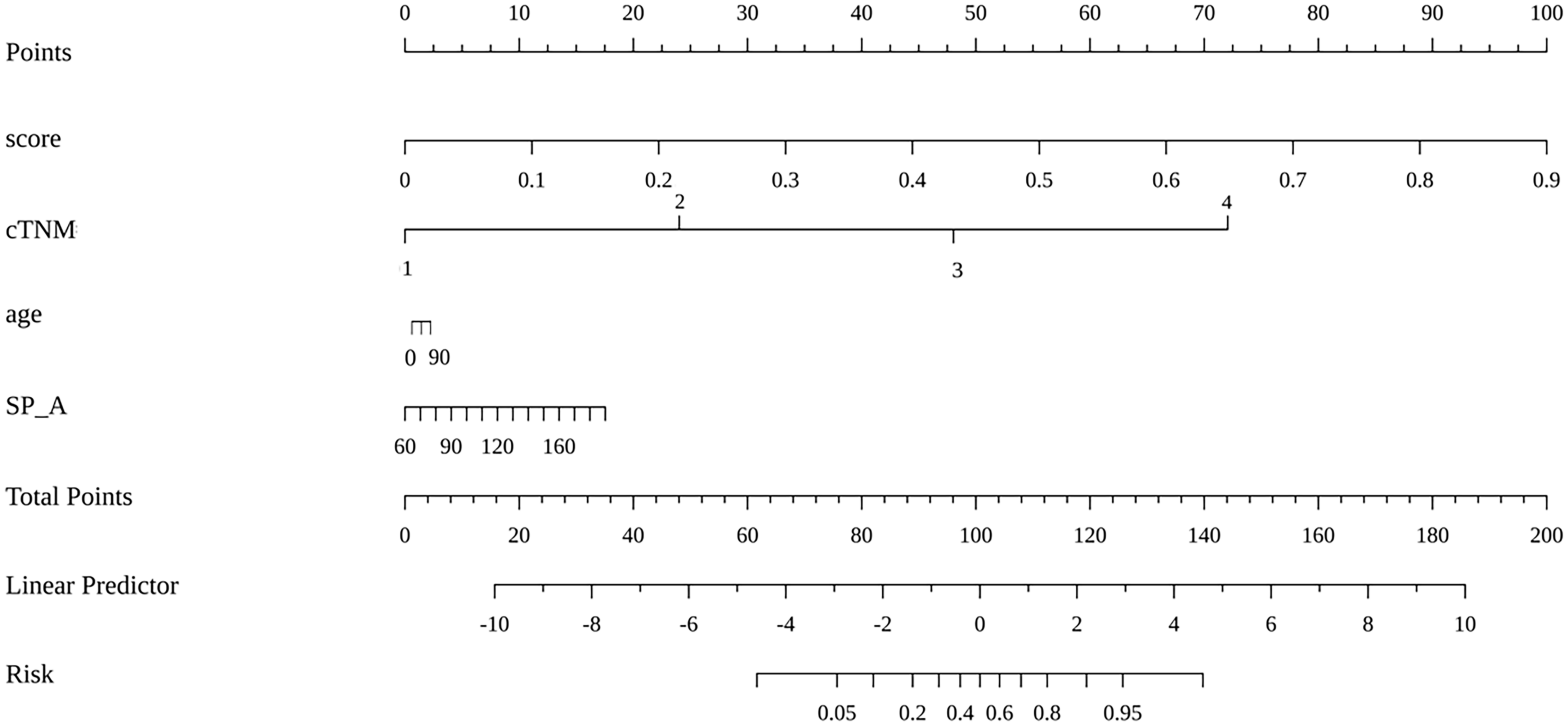
Radiomic nomogram based on radiomic signature and clinical factors.

**Figure 4:**
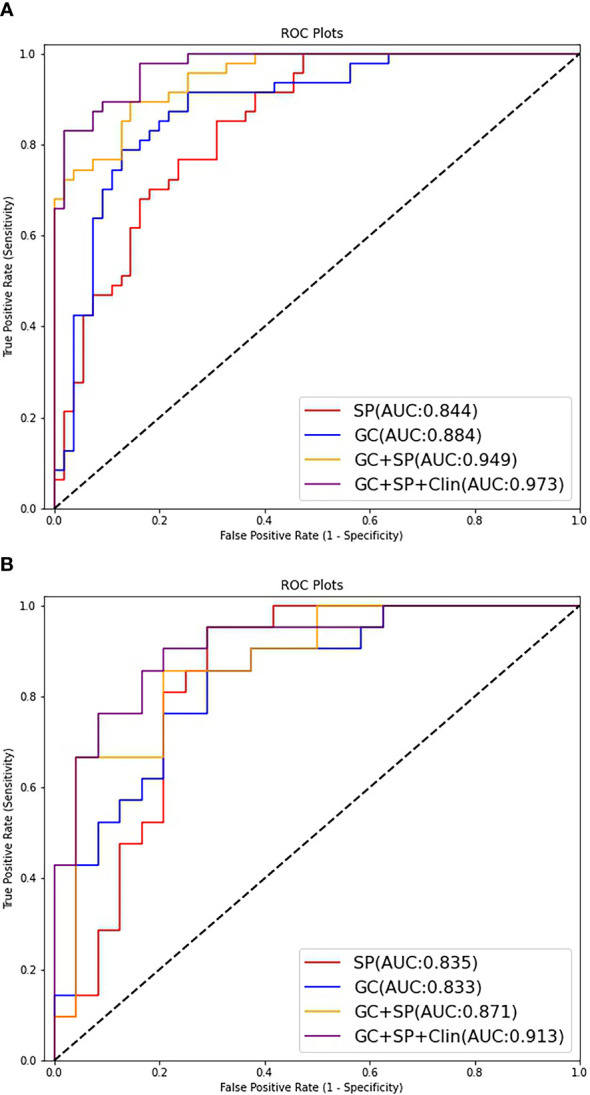
ROC curves of the different models for predicting differentiation state in the train cohort **(A)** and test cohort **(B)**.

**Figure 5:**
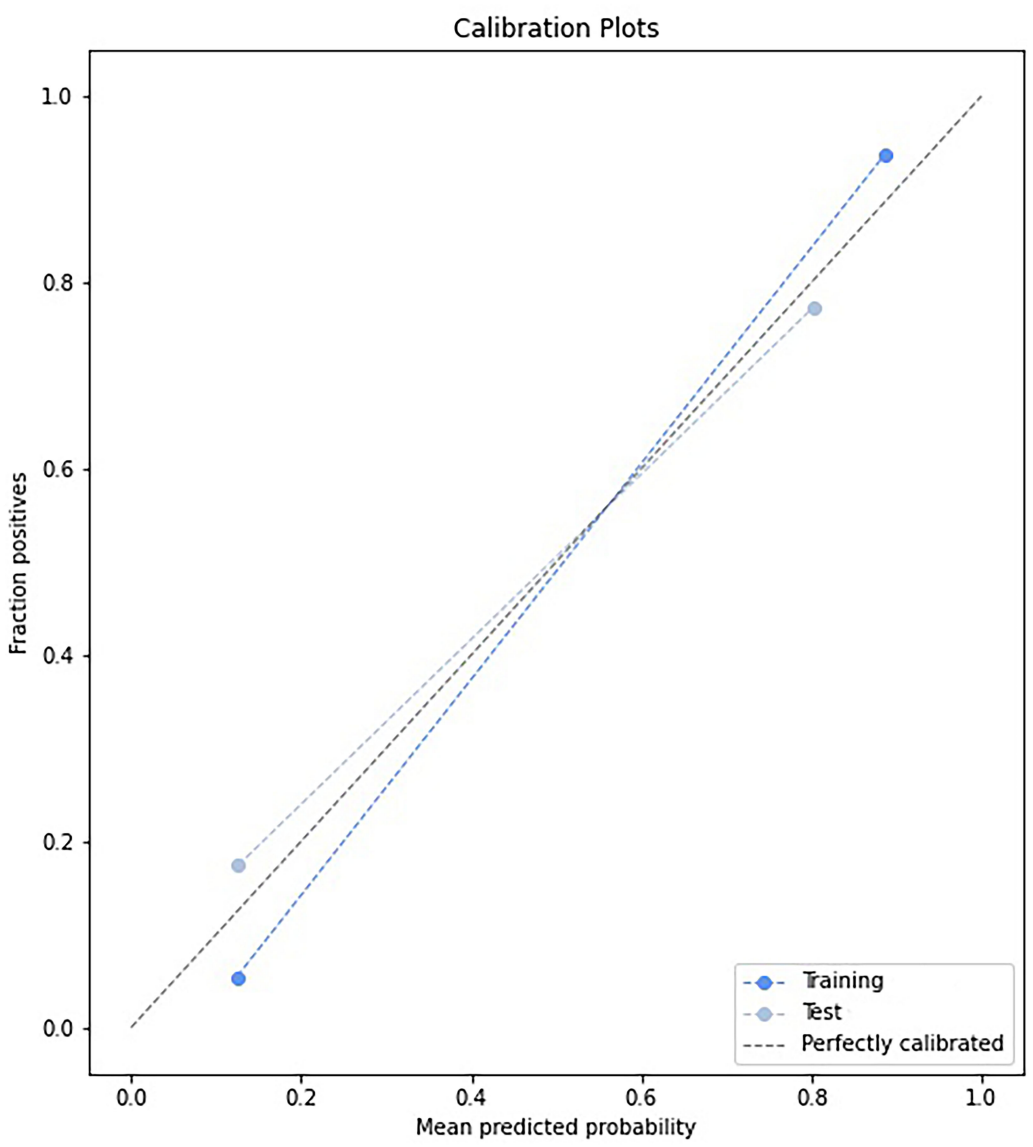
Calibration curve of the model (GC+SP+Clin).

The prediction model based on GC images, spleen images and the combination of two-stage images was developed and quantitatively integrated into three Radscores: GC Radscore, SP Radscore and GC+SP Radscore. As shown above, three clinical variables were remained as independent predictors after Univariate and multivariate logistic regressions. Furthermore, Logistic regression algorithm was used to yield CT–Based nomogram model (GC+SP+Clin model, **Table 2**; **Figure 3**). As shown in **Figure 4**, the developed nomogram exerted a powerful predictive ability in both training and test cohorts with AUCs of 0.97 [95% CI: 0.95, 0.99] and 0.91 [95% CI: 0.83, 0.99], respectively, which were higher than GC model (the radiomic signatures model of GC, **Figure 4**), SP model (the radiomic signatures model of spleen, **Figure 4**) and GC+SP model (incorporating GC and spleen radiomic signatures model in venous phase, **Figure 4**). To assess the possibility of overfitting, the Delong test was employed on the ROC curves of radiomics nomogram (GC+SP+Clin). The results indicated that the AUCs in test cohorts were not significantly different (*p*> 0.05). Furthermore, the Delong test was used for subgroup analysis of training set and test cohorts AUCs between different models (**Table 3**). The calibration curve indicated that the model demonstrated good agreement between the predicted probability and the expected probability (**Figure 5**).

**Table 2:** Selected radiomics features in GC+SP+C model.

Model	Selected features	Individual features	Coefficients
GC+SP+Clin	4		
		score	2.3889
		CT_SP_A	0.2811
		age	0.0452
		cTNM	1.2635

logit = 2.3889 x score + 1.2635 x cTNM + 0.0452 x age + 0.2811 x CT_SP_A + (-0.3404);

Radscore = 1/(1 + exp(-logit)).

GC+SP+Clin, gastric cancer, spleen and clinical combined model; CT_SP_A, CT attenuation of spleen in arterial phase; cTNM stage, clinical tumor node metastasis stage.

**Table 3:** Delong test results between models.

Group	SP *VS*. GC	SP *VS*. GC+SP	SP *VS*. GC+SP+Clin	GC *VS*. GC+SP	GC *VS*.GC+SP+Clin	GC +SP *VS*. GC+SP+Clin
Training cohort	0.380	0.000	0.000	0.030	0.007	0.037
Test cohort	0.980	0.660	0.515	0.460	0.380	0.677

The values in the **Table 4** represent the p values calculated by Delong test; GC+SP+Clin, gastric cancer, spleen and clinical combined; GC+SP, gastric cancer combined with spleen; GC, gastric cancer; SP, spleen.

Supplement material **Figure E3** showed the ROC curves of the testing group on each fold. The five-fold cross-validated AUCs of this model were 0.96, 0.92, 0.91, 0.90, and 0.88, which were all very close to the AUC of our combined model (GC+SP+Clin, AUC=0.91).

**Figure 6:**
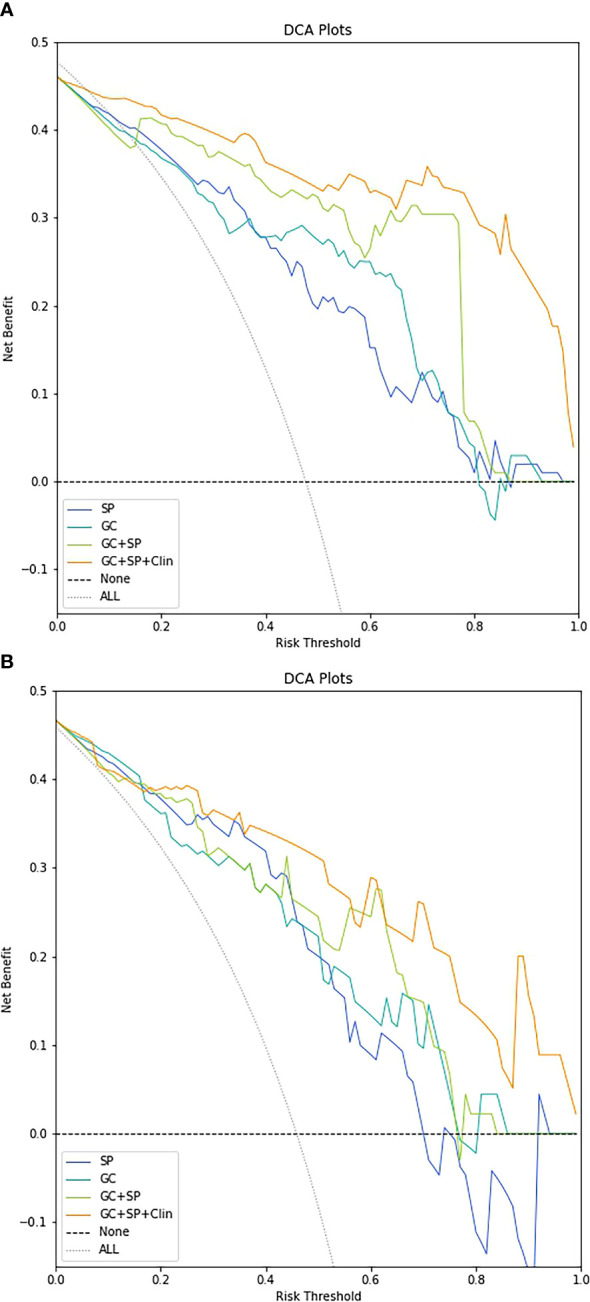
Decision-curve analysis for the different models in the train cohort **(A)** and test cohort **(B)**.

The decision curve showed relatively good performances for the GC+SP+Clin model compared with that for the GC model, SP model and GC+SP model. Across the majority of the range of reasonable threshold probabilities, the decision curve analysis showed that the GC+SP+Clin model had a higher overall benefit than the other models (**Figure 6**). In addition, the sensitivity, specificity, accuracy, F1-score and precision of different models in the diagnosis of GC differentiation are shown in **Table 4**. Finally, the sensitivity and specificity of enhanced CT were 53% and 51%, respectively.

**Table 4:** Comparison of the effectiveness of each model in identifying differentiation state in GC.

Model	Group	AUC	Accuracy	Sensitivity	Specificity	F1 Score	Precision
GC+SP+Clin	train cohort	0.97	87%	91%	84%	0.87	0.83
	test cohort	0.91	84%	86%	83%	0.84	0.82
GC+SP	train cohort	0.95	80%	89%	80%	0.84	0.79
	test cohort	0.87	77%	81%	79%	0.79	0.77
SP	train cohort	0.85	69%	77%	71%	0.73	0.69
	test cohort	0.84	74%	67%	79%	0.70	0.74
GC	train cohort	0.88	77%	85%	78%	0.81	0.80
	test cohort	0.83	75%	71%	79%	0.73	0.75

AUC, area under the curve; GC+SP+Clin, gastric cancer, spleen and clinical combined; GC+SP, gastric cancer combined with spleen; GC, gastric cancer; SP, spleen.

## Discussion

In this study, we investigated the value of splenic radiomic features in differentiating advanced gastric cancer with different degrees of differentiation. Preliminary analysis showed that the splenic radiomic features could distinguish the degree of AGC differentiation. On this basis, the value of tumor combined with spleen radiomic features in differentiating AGC differentiation degree was further studied. To quantify the value of spleen and tumor in differentiating poorly differentiated from non-poorly differentiated GC, we developed and tested a nomogram incorporating the radiomic features described above. Compared with general enhanced CT (sensitivity 53%, specificity 51%), the results have shown that the combined nomogram constructed by us can noninvasively stratify poorly differentiation and non-poorly differentiation GC, and it can efficiently identify patients with different degrees of differentiation, providing strong evidence for clinical treatment and personalized patient management.

In the present study, the various AGC histological types presented significant differences in their clinical and tumor features. And the five-year survival rates of patients with well differentiation, moderate differentiation and poorly differentiation were 87%, 57%, and 51%, respectively (2, 3). However, studies have shown that there are considerable histological differences between preoperative endoscopic biopsy and postoperative pathological results due to limitations such as sampling error, with 18% undifferentiated EGCs being diagnosed as differentiated type on initial biopsy histology (27). CT is a routinely used and easily accessible source of information in clinical oncology, and CT based radiomics has been widely used in the diagnosis and classification of GC, prognosis prediction and therapeutic effect evaluation (21, 24, 25). In our study, the radiomic characteristics of GC tumors were correlated with the differentiation status of GC, and the model was further established, which showed good performance in predicting the differentiation status of GC (the AUC of test cohort, 0.83). As a cavity organ, the delineation of tumor lesions is easily affected by the contents of the stomach, which will affect the extraction of radiomic features and reduce the accuracy of the model.

The spleen is closely associated with tumor development, and previous studies have shown that spleen features can predict tumor development (7, 16, 17, 28). Therefore, we studied the relationship between the radiomic characteristics of spleen and GC, and the results showed that the radiomic model established based on spleen was comparable to that of GC model, and the AUC was 0.84 [95% CI: 0.71, 0.96], with sensitivity was 67%, specificity was 79%. Consistent with the results of previous studies, spleen has important value in the diagnosis and treatment evaluation of GC (16, 17, 28). Studies have shown that dendritic cells, as powerful antigen presenting cells in the immune system, are closely related to the occurrence and development of tumors. However, dendritic cells in GC tissues were lower than those in normal tissues (11, 12). This also indicates that it is feasible to reflect the development of GC by the radiological features of spleen. In this study, we also confirmed in the training cohort that there was a statistical difference between the poorly differentiation and non-poorly differentiation groups in the splenic artery phase CT attenuation value and splenomegaly. Further univariate and multivariate analysis determined that the splenic artery phase CT attenuation value was an independent risk factor for predicting GC differentiation status (adjust OR=1.027, [95% CI: 1.00, 1.05], *p*=0.02).

Feng et al. showed that, compared with poorly differentiated GC, non-poorly differentiated GC tended to be elderly, male, small tumor size, shallow invasion depth, and less lymph node metastasis (2). Our results also confirm the results of Feng et al. In both the training cohort and the test cohort, there were statistical differences in age and cTNM stage between the two groups; in the training cohort (*p*<0.05), there were significant differences in cT and cN status between the two groups(*p*<0.05); In the test cohort, there were more males in the non-poorly differentiated group than in the poorly differentiated group(*p*<0.05). Further univariate and multivariate analysis of the clinical features of the training set showed that age (adjust OR=1.071, [95% CI: 1.02, 1.12], *p*=0.004) and cTNM stage (adjust OR=0.157, [95% CI: 0.06, 0.40], *p*=0.000) were independent risk factors for GC differentiation.

To sum up, we combined the radiomic features obtained above to establish a radiomic nomogram (GC+SP model). At the same time, the obtained clinical independent risk factors affecting the differentiation of GC were included to establish a combined nomogram (GC+SP+Clin model). And the developed nomogram exerted a powerful predictive ability in both training and test cohorts with AUCs of 0.97 [95% CI: 0.95, 0.99] and 0.91 [95% CI: 0.83, 0.99], respectively, which were higher than other models. Furthermore, the results of five-fold cross validation also show that the final established combined model has good stability. In addition, the decision curve showed that the GC+SP+Clin model had a higher overall benefit than the other models. This result is also consistent with previous studies. The constructed combined model contains more characteristic parameters, which can improve the prediction efficiency of the model (7, 22, 27).

There are several limitations in this study. First of all, as a single-center, small-sample study, we lack external validation, so we need to cooperate with other institutions to conduct a multi-center, large-sample study. Second, no further survival analysis and clinical efficacy study were conducted, because this study was only a preliminary exploration, and other relevant studies will be further carried out in the follow-up study. Finally, only the maximum and upper and lower layers were sketched for the ROI of the spleen, so the key points of spleen volume mentioned in other studies were not included (7, 17), which will be supplemented in the following studies. In addition, serological immune indicators and tumor markers included in relevant studies were not included in the final model establishment of this study (16, 28–30), which may be related to the small sample size. Therefore, further studies with more detailed sample size need to be carried out.

## Conclusion

This study provides valuable insights into splenic CT image-based determinants of differentiation status in GC. In addition, we proposed and validated a Nomogram based on spleen and GC for inferring the state of differentiation in GC. We found that the Nomogram has good predictive power and can be used as an easy-to-implement, non-invasive and practical quantitative tool for predicting the state of differentiation in GC. In short, nomogram based on spleen combined with GC helps to identify GC patients with different differentiated states, so that they can benefit from treatment.

## Data availability statement

The original contributions presented in the study are included in the article/**Supplementary Material**. Further inquiries can be directed to the corresponding author.

## Ethics statement

The studies involving human participants were reviewed and approved by The Institutional Review Board of the first affiliated Hospital of Zhengzhou University (No. 2021-KY-1070-002). Written informed consent for participation was not required for this study in accordance with the national legislation and the institutional requirements. A waiver of informed consent was obtained.

## Author contributions

All authors contributed to the study conception and design. Material preparation, data collection and analysis were performed by DL, PL, XC, MC, BZ, ML, and CH. The first draft of the manuscript was written by DL, PL, and XC, and all authors commented on previous versions of the manuscript. All authors contributed to the article and approved the submitted version.
