# Supplementary material for: Are radiomic spleen features useful for assessing the differentiation status of advanced gastric cancer?
Source: Front Oncol. 2023 May 5;13:1167602. doi: 10.3389/fonc.2023.1167602 (PMC10196477; doi:10.3389/fonc.2023.1167602)
Supplement: Supplementary file 1 [file DataSheet_1.docx]

**Appendix E1**

**Materials and Methods**

***Data collection***

Laboratory analysis of immune-related serological indicators was performed via routine blood tests within 1 week before surgery. Positive lymph nodes were defined as short-axis diameter >10 mm or presented with heterogeneous enhancement, and patients with detected positive lymph nodes on CT images were considered as CT-reported-LN positive (1,2,3). Gastric tumors were analyzed by Bormann classification depending on tumor size, infiltration and development of ulcers (2,3). The cT stage and cTNM stage were classified according to the eighth edition of the Cancer Staging Manual of the American Joint Committee on Cancer (AJCC)/International Union Against Cancer (UICC) staging system (4,5). The maximum tumor thickness was measured according to the maximal short diameter perpendicular to the long axis of the tumor maximum cross-sectional area. At the same time, the CT attenuations of the tumor were measured at the maximum cross-sectional area (unenhanced phase, arterial phase and venous phase), avoiding necrotic areas. At the splenic hilar level cross-sectional area, the spleen thickness was measured. The CT attenuations of the spleen were measured at the splenic hilar level cross-sectional area (unenhanced phase, arterial phase and venous phase), avoiding blood vessels and calcification. All the CT attenuation and thickness were taken three times, and the mean value was calculated for the next statistical analysis.

***Image acquisition and segmentation***

A conventional axial scan (120 kV, 350 mA, a field of view = 500 mm, matrix 512×512, and section thickness 5 mm) was performed before and after intravenous injection of non-ionic iohexol (iopromide, 370 mg/mL, GE Medical Systems, 1.5 mL/kg and 3 mL/s) by a dual-head pump injector (Medrad, Warrendale, USA). Finally, 20 mL of saline flush was injected at a rate of 3 mL/s. Contrast-enhanced CT scans were performed with a scanning delay of 30 s (arterial phase) and 70 s (venous phase) after the start of intravenous (i.v.) injection of iopromide.

Tumor segmentation was performed by manual whole tumor segmentation at the cross-sectional area, with care taken to avoiding obvious necrosis, vessels, calcification, and cystic portions. The intraluminal fluid or gas were also manually excluded. An example of the tumor segmentation is presented in Supplementary material Figure E1. Splenic segmentation was performed at the three selected contiguous sections at the splenic hilar level cross-sectional area, with care taken to avoiding vessels and calcification. An example of the spleen segmentation is presented in Supplementary material Figure E2. Any disagreement was resolved through discussion.

**Results**

***Nomogram construction and evaluation***

The Supplement material Figure E3 showed the ROC curves of the testing group on each fold. The values of AUC of different folds were all very close to the AUC of our combined model (AUC=0.91), which indicated good stability of our combined model.

**References**

1. Dong D, Tang L, Li ZY, et al. Development and validation of an individualized nomogram to identify occult peritoneal metastasis in patients with advanced gastric cancer. *Ann Oncol* (2019) 30:431-8. doi: 10.1093/annonc/mdz001.

2. Li J, Fang M, Wang R, et al. Diagnostic accuracy of dual-energy CT-based nomograms to predict lymph node metastasis in gastric cancer. *Eur Radiol* (2018) 28:5241-9. doi: 10.1007/s00330-018-5483-2.

3. Zheng Z, Zhang Y, Zhang L, et al. A nomogram for predicting the likelihood of lymph node metastasis in early gastric patients. *BMC Cancer* (2016)16: 92. doi: 10.1186/s12885-016-2132-5.

4. Brierley JDGM,Wittekind C, et al., editor. TNM Classification of Malignant Tumours, 8th edn. *wiley Blackwell: Union for International Cancer Control (UICC)*, 2017. 2.

5. Amin MBES, Greene F, Byrd DR, et al., editor. AJCC Cancer Staging Manual, 8th edn. New York, NY, USA: *Springer International Publishing*, 2017.

**Table E1: Multivariate Logistic Regression Analysis for the Clinical Characteristics and CT Parameters**

| **Variable** | **β** | **z** | ***p*** | **Adjusted OR** | **95% CI** |
| --- | --- | --- | --- | --- | --- |
| **Intercept** | -2.575 |  |  |  |  |
| **Age** | 0.069 | 2.846 | 0.004* | 1.071 | 1.022 ~ 1.123 |
| **cTNM stage** | -1.849 | -3.884 | 0.000* | 0.157 | 0.062 ~ 0.400 |
| **CT_SP_A** | 0.026 | 2.333 | 0.02* | 1.027 | 1.004 ~ 1.049 |

* *p*<0.05, cTNM stage,clinical tumor node metastasis stage; CT, computed tomography; OR, odds ratio; CT_SP_A, CT attenuation of spleen in arterial phase.

| **A 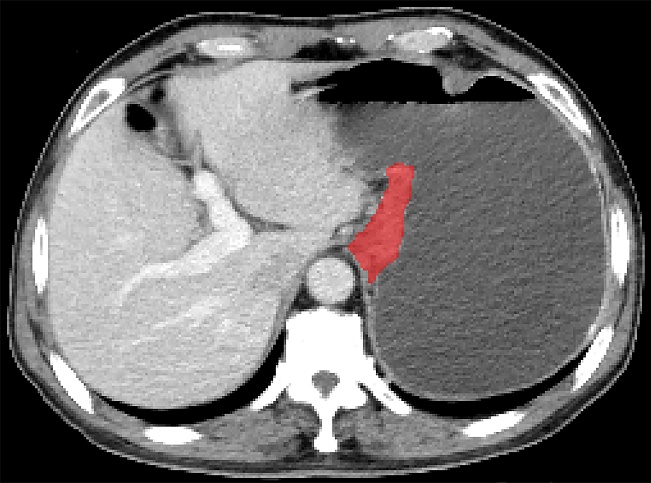** | **B 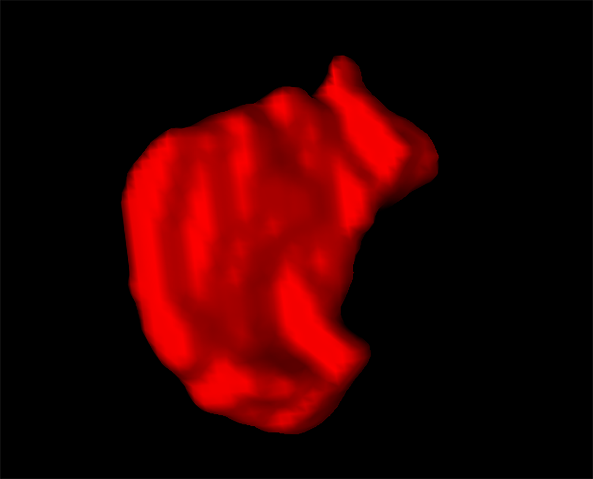** |
| --- | --- |

**FIGURE E1:** An example of the GC segmentation. **(A)** The venous phase of the tumor was delineated on CT images of interest. **(B)** Tumor reconstruction image of ITK SNAP.

| **A 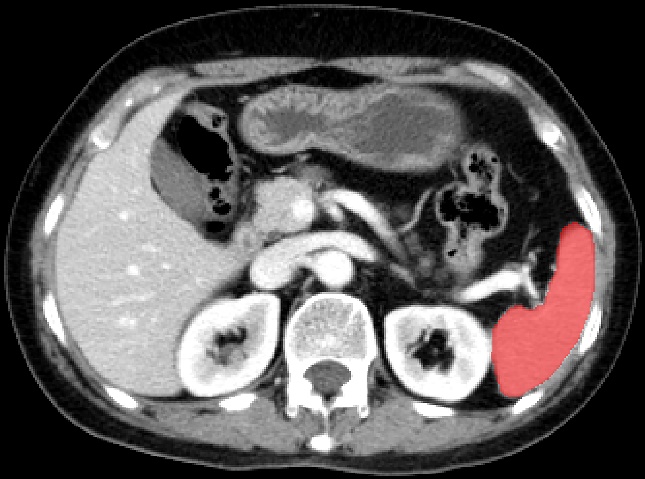** | **B 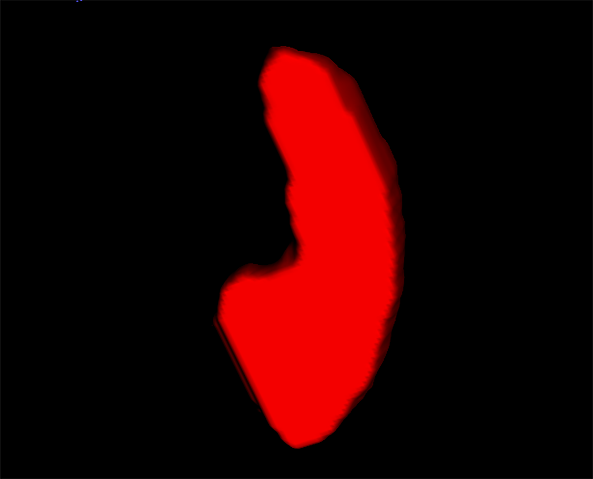** |
| --- | --- |

**FIGURE E2:** An example of the spleen segmentation. **(A)** The venous phase of the spleen was delineated on CT images of interest. **(B)** Spleen reconstruction image of ITK SNAP.


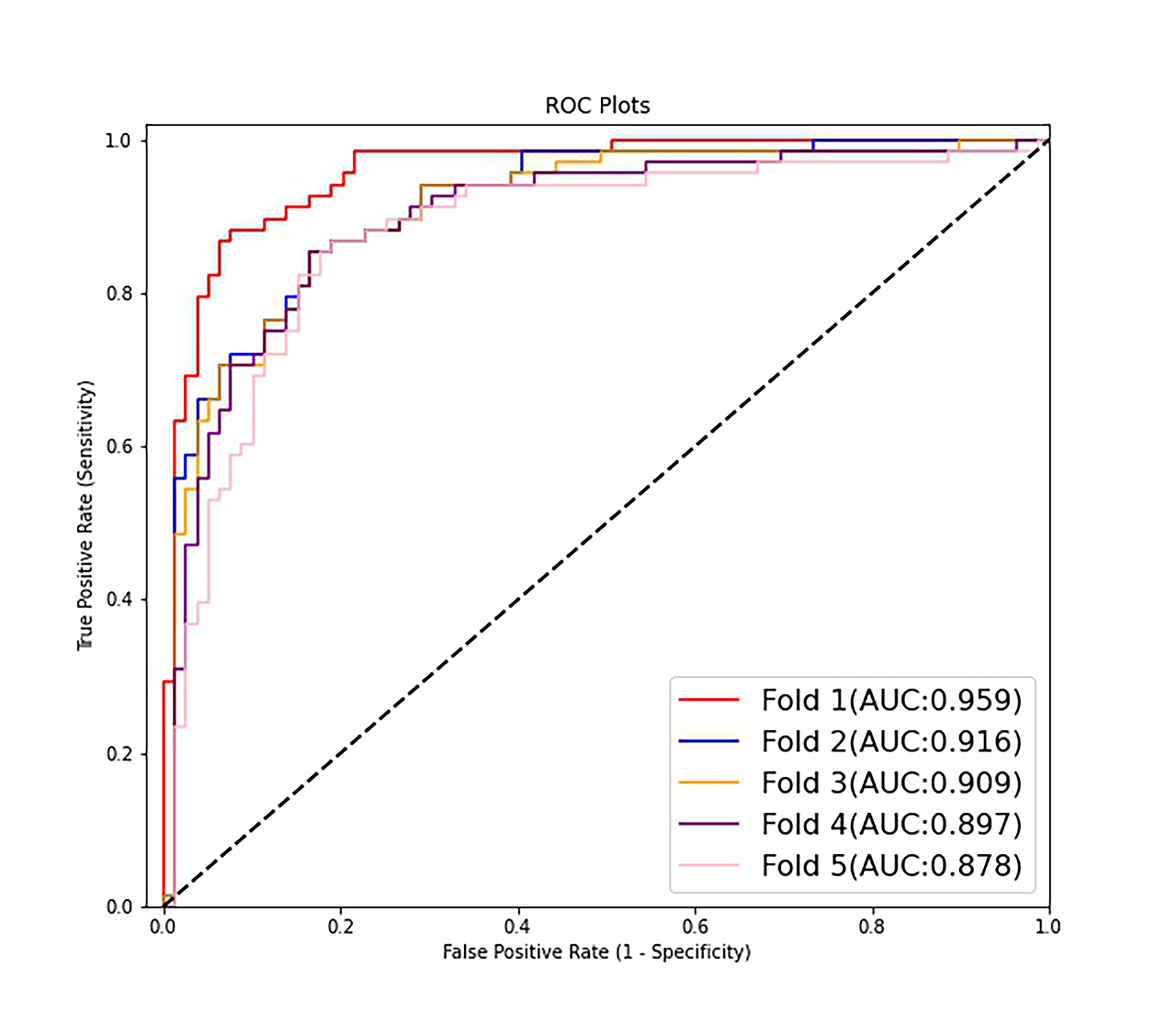


**FIGURE E3:** The values of AUC in five-fold cross validation.
